# Importance of T1-Mapping Sequence in Patients with Hypertrophic Cardiomyopathy without Foci of Non-Ischemic Myocardial Injury in Late Gadolinium Enhancement Sequence

**DOI:** 10.3390/biomedicines12061330

**Published:** 2024-06-14

**Authors:** Natalia Zdebik, Rafał Poręba, Paweł Gać

**Affiliations:** 1 Centre for Diagnostic Imaging, 4th Military Hospital, 50-981 Wroclaw, Poland; 2 Department of Internal Medicine, Occupational Diseases, Hypertension and Clinical Oncology, Wroclaw Medical University, 50-556 Wroclaw, Poland; 3 Department of Population Health, Division of Environmental Health and Occupational Medicine, Wroclaw Medical University, 50-345 Wroclaw, Poland

**Keywords:** cardiac magnetic resonance, extracellular volume, hypertrophic cardiomyopathy

## Abstract

Background: The aim of this study was to assess the importance of T1-mapping sequences in the diagnosis of hypertrophic cardiomyopathy (HCM) in patients without foci of non-ischemic myocardial injury in classic cardiac magnetic resonance (CMR) sequences. Methods: Two groups were compared: 28 patients with HCM, without any foci of myocardial injury in the late gadolinium enhancement (LGE) sequence (HCM group), and 28 patients without cardiomyopathy (CON group). Classic CMR sequences and T1-mapping sequences were performed. The following parameters were assessed: T1 time of the whole left ventricular myocardium, T1 time of myocardium in the basal, middle and apical layers of the left ventricle, and T1 time in individual segments of the left ventricular myocardium. Myocardial extracellular volume (ECV) was assessed similarly. Results: ECV was significantly higher in the HCM group than in the CON group, for the whole left ventricular myocardium, for the basal and apical layers of the left ventricle, and for segments 1–3, 8, and 13–16 of the left ventricle. Regression analysis showed that a higher left-ventricular mass index (LVMI), a higher body mass index and older age are factors independently associated with a higher ECV of the whole myocardium but only in the group with LVMI ≥ 131.84 g/m^2^. Conclusion: In patients with HCM without foci of non-ischemic myocardial injury, higher ECV values of the left ventricular myocardium are observed.

## 1. Introduction

Hypertrophic cardiomyopathy (HCM), with a prevalence of 1:500 in the general population, is a relatively common autosomal-dominant inherited monogenic disorder. HCM is characterized by left ventricular (LV) hypertrophy, which is most often asymmetric. Histologically, left ventricular hypertrophy is associated with the disruption of the myocardial fiber system, coronary microcirculation dysfunction, and myocardial replacement fibrosis. Myocardial fibrosis in HCM is a poor prognostic factor [1,2]. Myocardial hypertrophy most often affects the interventricular septum. However, any segment of the myocardium may also be hypertrophied [1].

Left ventricular wall thickness is 15 mm or more according to the European Society of Cardiology guidelines (the average is 21 mm) but may demonstrate massive thickness (30 to 50 mm) in some cases [3]. However, normal dimensions may occur in gene carriers [4].

Clinical symptoms encompass cardiac-related symptoms like dyspnea, chest pain, palpitations and syncope, or asymptomatic signs like a heart murmur or an abnormal electrocardiogram [5,6]. The most common cardiovascular events in patients with HCM include sudden cardiac death (51%), heart failure (36%), and stroke (13%) [6,7].

Clinically, hypertrophic cardiomyopathy is diagnosed based on a confirmation of myocardial hypertrophy of the non-enlarged LV using diagnostic imaging, correlating with anamnesis data [4]. The basic diagnostic imaging method in the assessment of HCM is echocardiography due to its good availability and low costs. The routine use of cardiac magnetic resonance (CMR) in the management of HCM patients has recently become the standard [8].

Late gadolinium enhancement (LGE) is a reference standard for the non-invasive imaging of myocardial scar and focal fibrosis. Typically, in hypertrophic cardiomyopathy, foci of non-ischemic myocardial injury (intramural or subepicardial LGE foci) are found. Topographically, they most often occur first in hypertrophied segments of the myocardium of the left ventricle, as well as in RV–LV insertion points. The occurrence of LGE foci in patients with hypertrophic cardiomyopathy is an unfavorable prognostic factor [9]. Gadolinium contrast media cannot cross intact sarcolemma membranes [6]. LGE sequences can detect only regional myocardial fibrosis [10].

T1 mapping measures the longitudinal or spin-lattice relaxation time [2,9]. Gadolinium-based contrast agents are distributed throughout the extracellular space and shorten T1 relaxation times of the myocardium proportional to the local concentration for gadolinium. Pre- and post-contrast T1-mapping techniques are used to quantify myocardial extracellular volume (ECV). HCM patients have significantly shorter post-contrast myocardial T1 times compared to healthy controls due to myocardial elevated diffuse fibrosis [2,11].

Native T1 values are a composite signal of myocytes and extracellular volume that decrease in fat or iron deposition in the myocardium while increasing with edema, amyloid deposition, and fibrosis. Studies proved that native (non-contrast) T1 mapping can differentiate myocardial abnormality of hypertrophic cardiomyopathy from healthy myocardium [12]. T1-mapping sequences can also be used in the differential diagnosis of myocardial accumulation diseases, e.g., in patients with amyloidosis. In AL amyloidosis, due to the predominant myocardial edema, very high values of myocardial T1 time and moderately high ECV values are observed. However, in ATTR amyloidosis, which is dominated by amyloid deposition in the myocardium, very high ECV values are observed with moderately high myocardial T1 times [13]. T1-mapping sequences may also be useful in monitoring the course of the disease. T1-mapping sequences enable the quantitative measurement of the absolute T1 time of the myocardium. Therefore, it is possible to compare the values of the measured T1 time and ECV between subsequent acquisitions and assess the dynamics of changes occurring in the myocardium [13].

T1-mapping imaging is currently used in cardiac magnetic resonance (CMR) protocols in clinical practice. However, most scientific evidence regarding the usefulness of T1 mapping concerns patients with foci of non-ischemic myocardial damage in the LGE sequence. There is less scientific evidence regarding the importance of T1-mapping sequences in patients without myocardial damage in the LGE sequence.

The aim of this study was to assess the importance of T1-mapping sequences in the diagnosis of hypertrophic cardiomyopathy in patients in whom no signs of focal myocardial injury were found using classic CMR imaging sequences.

## 2. Materials and Methods

### 2.1. Study Group

The study was conducted in accordance with the Declaration of Helsinki and approved by the Ethics Committee of Wroclaw Medical University (protocol code KB-414/2021). Informed consent was obtained from all subjects involved in the study.

The study protocol included estimating the required size of the study group, qualifying patients to the group with hypertrophic cardiomyopathy without LGE foci (HCM group) based on the assumed inclusion and exclusion criteria, and selecting patients for the control group (CON group).

The size of the study group was estimated based on the sample size calculator. The following evaluation conditions were assumed in the calculator: estimated fraction size 5%, significance level 0.05, population size 2,800,000, and permissible error 10%. The minimum required size of the study group was estimated at 19 patients.

The criteria for inclusion in the HCM group were age >18 years, diagnosis of hypertrophic cardiomyopathy based on the diagnostic procedure (wall thickness >15 mm or septal to lateral wall thickness ratio >1.3 or apical to basal wall thickness ratio ≥1.3 to 1.5 in apical HCM), and consent to participate in the study. The criteria for exclusion from the study in the initially recruited HCM group were the presence of non-ischemic myocardial injury (LGE foci) on cardiac magnetic resonance imaging, other causes of hypertrophic left ventricular morphotype in CMR (amyloidosis, sarcoidosis, athlete’s heart, hypertensive cardiomyopathy, aortic stenosis, third degree obesity, systemic diseases), lack of hypertrophic left ventricular morphotype in CMR, insufficient technical quality of the examination CMR, and incidentally diagnosed ischemic heart disease with ischemic myocardial injury in CMR. Based on the inclusion criteria, a group of 69 patients was included in the study, and 41 patients were excluded from the study group.

The CON group was recruited from healthy volunteers with similar anthropometric parameters (same gender, age ±3 years, BMI ±2 kg/m^2^). The health status of the volunteers was verified based on interviews, physical examinations, echocardiography and the analysis of the results of cardiac magnetic resonance performed in the project.

Finally, 2 groups of subjects were compared: 28 patients with hypertrophic cardiomyopathy, without any foci of myocardial injury in the late gadolinium enhancement (LGE) sequence (HCM group; mean age 52.17 ± 6.35 years), and 28 patients without cardiomyopathy (CON group; mean age 51.76 ± 6.49 years). Basic clinical information about the study group is summarized in Table 1.

### 2.2. Cardiac Magnetic Resonance

**Figure 1:**
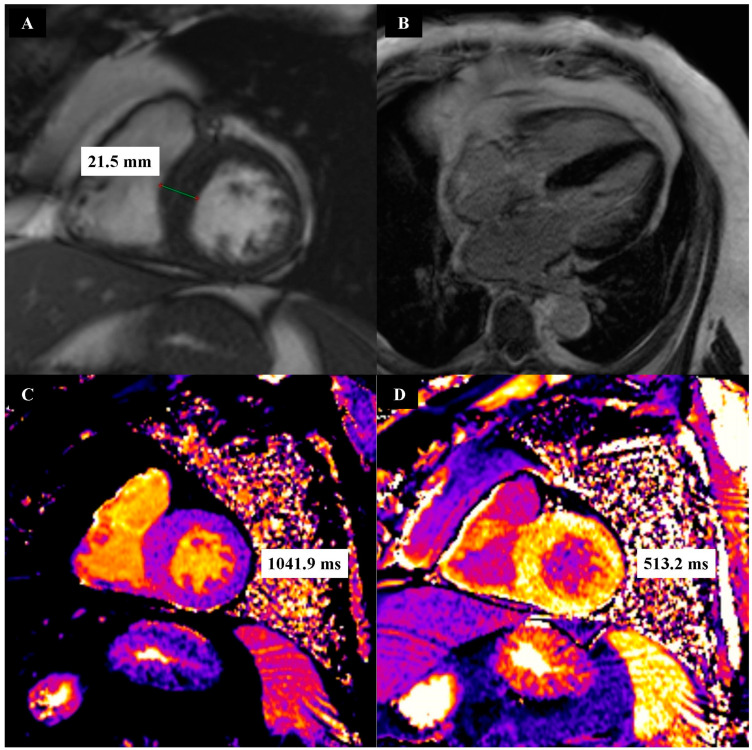
Example CINE (**A**), late gadolinium enhancement (**B**), native T1 mapping (**C**) and post-contrast T1 mapping (**D**) images in patients from the hypertrophic cardiomyopathy group.

**Figure 2:**
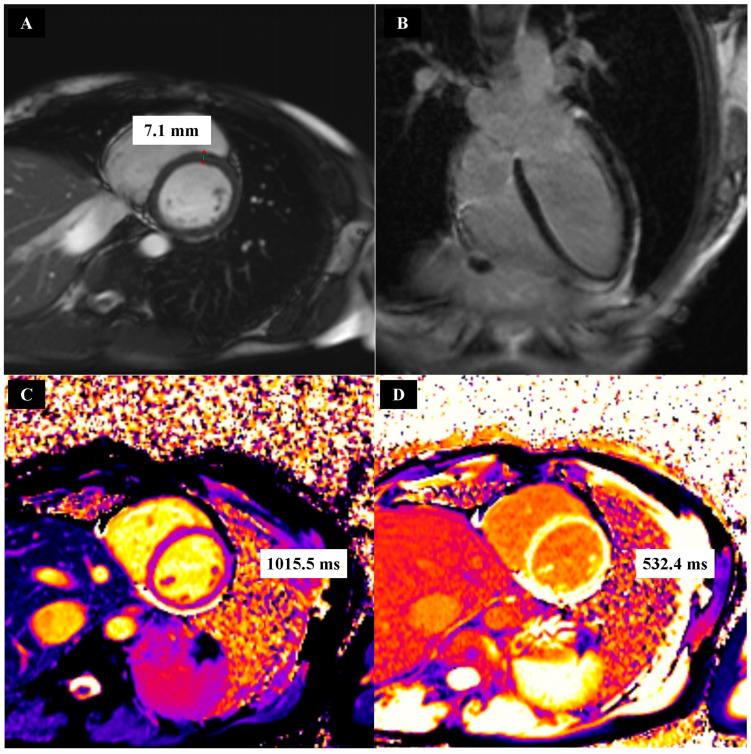
Example CINE (**A**), late gadolinium enhancement (**B**), native T1 mapping (**C**) and post-contrast T1 mapping (**D**) images in patients from the control group.

First, 1.5 Tesla cardiac magnetic resonance (CMR) imaging was performed using the Magnetom Aera device (Siemens Healthcare, Forchheim, Germany). All CMR was performed using the same examination protocol. This protocol consisted of a steady-state slow precession CINE sequence, a short tau inversion recovery (STIR) sequence, a native T1-mapping sequence, a late gadolinium enhancement (LGE) sequence, and a T1-mapping C+ sequence. T1-mapping C+ sequences were performed 5 min and 20 min after intravenous bolus injections of gadobutrol (Gadovist, Bayer Healthcare, Leverkusen, Germany), at a dose of 0.2 mmol/kg body weight. Medis Suite MR 4 software (Medis, Leiden, The Netherlands) was used to evaluate the post-processing of CMR images. Examples of CMR images obtained for a patient from the HCM group are shown in Figure 1, and the same can be seen for a patient from the CON group in Figure 2.

CINE sequences were obtained in the left ventricular short axis and long axis in two-chamber, three-chamber, and four-chamber views. The following linear and planimetric measurements of the cardiac chambers were conducted based on CINE sequence images: right atrial area (RAA), left atrial area (LAA), left ventricular end-diastolic diameter (LVEDD), left ventricular end-systolic diameter (LVESD), anterior interventricular septal diastolic diameter (aIVSDD), posterior wall diastolic diameter (PWDD) and maximum left ventricular myocardial diastolic diameter (maxLVMDD).

CINE sequence images were also used to measure left ventricular functional parameters. End-diastolic and end-systolic volumes (EDV and ESV) were estimated using the volumetric method. EDV and ESV values were calculated as the sum of the left ventricular area in successive short-axis image layers multiplied by the slice thickness in the left ventricular end-diastolic and end-systolic phases, respectively. The difference between EDV and ESV values was the stroke volume (SV), and the percentage ratio of SV to EDV was the ejection fraction (EF). All left ventricular functional parameters were expressed as values indexed by body surface area (BSA). In addition, the left ventricular mass (LVM) and left ventricular mass index (LVMI) were estimated.

In the STIR sequence, the occurrence of foci of left ventricular oedema was assessed, and in the LGE sequence, the occurrence of foci of left ventricular myocardial injury was assessed.

In addition to classical sequences, T1-mapping sequences were performed. The following parameters were assessed: T1 time of the whole left ventricular myocardium (T1 whole myocardium), T1 time of the myocardium in the basal (T1 basal), middle (T1 middle) and apical (T1 apical) layers of the left ventricle, and T1 time in individual segments of the left ventricular myocardium. The T1 time for the whole left ventricular myocardium is the average of the T1 time of all myocardium voxels. The T1 time for each of the left ventricular myocardial layers is the average of all voxels in each of these layers. The T1 time for each of the 16 left ventricular myocardial segments is the average of all voxels in each of these segments. Myocardial extracellular volume (ECV) was assessed similarly. Hematocrit for ECV calculation was determined in the range of 0–48 h before the study.

Details of the CMR protocol are presented in the center’s previous publication [14].

### 2.3. Statistical Analysis

Dell Statistica (data analysis software system), version 13 (Dell Inc., Tulsa, OK, USA), was used for statistical calculations. Quantitative variables were presented as arithmetic means ± standard error of mean. Due to the high probability of type II statistical errors, quantitative variables were compared using one-way MANOVA. In the first stage, it was assessed whether there was a contrast in any of the assessed variables, obtaining the value of the Wilks Lambda statistic with the appropriate *p*-value. In the case of the statistical significance of the Wilks Lambda statistic, the impact of the contrast of the compared factor (HCM vs. CON) was estimated for each of the analyzed variables. Qualitative variables were presented as percentages. Percentage distributions of qualitative variables were compared using the maximum likelihood chi-square test. Correlation analysis and regression analysis were used to assess the relationship between variables. In the case of quantitative variables with a normal distribution, Pearson’s r correlation coefficients were determined. In the case of quantitative variables with a distribution other than normal, Spearman’s r coefficients were determined. Results at the *p* < 0.05 level were considered statistically significant.

## 3. Results

In CINE sequences of the HCM group compared to the CON group, the anterior interventricular septal diastolic diameter and posterior wall diastolic diameter were significantly higher. The maximum left ventricular myocardial diastolic diameter, left ventricular mass and left ventricular mass index were also statistically significantly higher in the HCM group than in the CON group. In the HCM group, thickening of the basal segments of the interventricular septum and the anterior wall of the left ventricle; thickening of the middle segments of the interventricular septum; thickening of the apical segments of the interventricular septum, inferior and lateral walls of the left ventricle; and apex thickening occurred significantly more often in the HCM group than in the CON group, as seen in Table 2.

There was no difference in the left ventricular ejection fraction between the HCM and CON groups. Left ventricular systolic dysfunction was not observed in both study groups, as seen in Table 2.

In the classical sequences of the structural assessment of the LV myocardium, i.e., in the STIR and LGE sequences, which resulted from the inclusion and exclusion criteria, no statistically significant differences were observed either, as shown in Table 2.

Myocardial T1 times did not differentiate the studied groups. No differences were observed in the mean values of the T1 time of the whole myocardium, the mean values of the T1 time of the subsequent layers of the left ventricle—the basal, middle and apical layers, or the mean values of the T1 times of each of the 16 left ventricular myocardial segments (Table 3).

**Figure 3:**
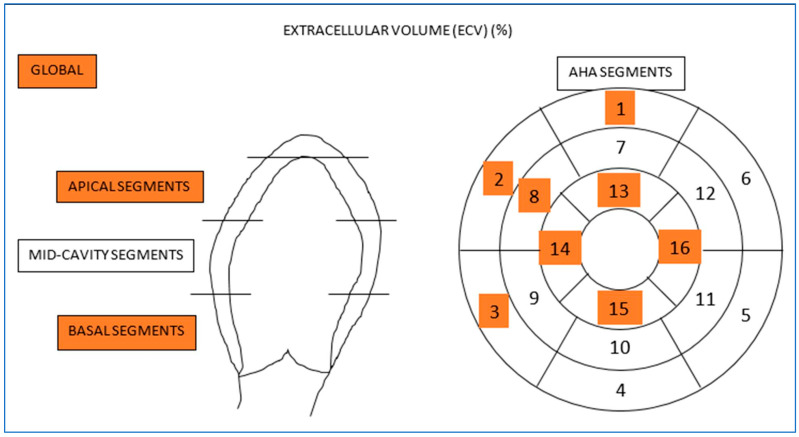
Location of significantly higher extracellular volume values in the left ventricular myocardium in patients with hypertrophic cardiomyopathy without foci of myocardial injury in the late gadolinium enhancement sequence. Statistically significant differences regarding the entire myocardium, myocardial layers and myocardial segments are marked in orange. AHA—American Heart Association.

However, the study groups differed in the extracellular volume of the myocardium. In the HCM group, compared to the CON group, the ECV values of the whole myocardium, the ECV values of the basal and apical ECV, and the values of ECV segments 1–3, 8, and 13–16 were statistically significantly higher, as seen in Table 4, Figure 3.

The performed correlation analysis showed no linear relationship between left ventricular myocardial thickness (aIVSDD, PWDD, maxLVMDD) and T1 times and extracellular volumes of the left ventricle myocardium. There were also no significant linear relationships between left ventricular mass (LVM, LVMI) and myocardial T1 times and ECV in the T1-mapping sequence (Table 5).

Based on the multivariate segmental linear regression analysis with a break-through point carried out for the entire study group, the following relationship model was obtained:

- For LVMI < 131.84 g/m^2^: whole myocardium ECV = 15.528 + 0.025 LVMI + 0.259 BMI + 0.124 age (*p* > 0.05).
- For LVMI ≥ 131.84 g/m^2^: whole myocardium ECV = 24.025 + 0.038 LVMI + 0.271 BMI + 0.139 age (*p* < 0.05).

The obtained model indicates that in the study group, a higher LVMI, higher BMI and older age are factors independently associated with higher extracellular volume values of the whole myocardium but only in the group of patients with LVMI ≥ 131.84 g/m^2^. At lower LVMI values, the relationship between the LVMI and ECV of the whole myocardium becomes statistically insignificant (Table 6).

## 4. Discussion

HCM is characterized by the excessive thickening of the ventricular myocardium, particularly the basal interventricular septum subjacent to the aortic valve [5,15]. Cardiac hypertrophy may be occasionally restricted to other myocardial regions [3]. In our study, the end-diastolic thickness of the anterior part of the interventricular septum and the end-diastolic thickness of the posterior wall of the left ventricle were significantly higher in the HCM group compared to the CON group. Also, the left ventricular mass index was significantly higher in the HCM group than in the CON group. However, increased left ventricular mass is not a requirement for establishing a clinical diagnosis of hypertrophic cardiomyopathy. According to some studies, the LV mass index was normal in approximately 20% of patients with a specific HCM phenotype [16]. In the study group, a higher LVMI, higher BMI and older age were factors independently associated with higher ECV values of the whole myocardium but only in the group of patients with LVMI ≥ 131.84 g/m^2^ (at lower LVMI values, the relationship between the LVMI and ECV of the whole myocardium becomes statistically insignificant). The LVMI is a recognized independent predictor of adverse cardiovascular events in patients with HCM, such as mortality, need for heart transplantation, and the occurrence of malignant ventricular arrhythmia [17]. ECV is a biomarker of diffuse interstitial fibrosis. It has been postulated that extracellular volume and the LVMI did not correlate with each other [18].

Left ventricular systolic function is characteristically normal to hyperdynamic in patients with hypertrophic cardiomyopathy, which was proved in this study, because both groups did not differ in left ventricular ejection fraction [3]. However, previous studies suggested that 4–9% of patients develop systolic dysfunction, defined by an LV ejection fraction (LVEF) < 50%. This complication of disease has previously been termed end-stage or burnt-out HCM and is typically accompanied by diffuse myocardial fibrosis (although left ventricular wall thinning and cavity enlargement may also be present) [19]. The left ventricle in hypertrophic cardiomyopathy usually has a normal end-diastolic volume and a reduced end-systolic volume [3]. In this study, groups did not differ in left ventricular end-systolic and end-diastolic diameter. The studied groups also did not differ in right and left atrium surface area in four-chamber projection. Left atrial volume is often increased in patients with hypertrophic cardiomyopathy and is a known predictor of the development of atrial fibrillation and heart failure [3]. An increased left atrial volume in HCM is associated with greater hypertrophy, more diastolic dysfunction, and higher filling pressures. From a clinical point of view, left atrial anteroposterior diameter is a well-known marker of HCM severity [20].

The non-invasive assessment of diffuse interstitial fibrosis is of interest in terms of better risk stratification in HCM patients [21].

The presence of late gadolinium enhancement in cardiac MRI in hypertrophic cardiomyopathy reflects irreversible myocardial injury and is a significant prognostic factor in the prediction of severe cardiac complications [7,22]. Extensive LGE (15% of LV mass) can identify HCM patients with increased risk for sudden cardiac death and progressive heart failure [21]. In our study, there were no patients with focal myocardial damage on the LGE sequence. According to available studies, higher ECV values are observed in HCM patients with focal LV myocardial damage in the LGE sequence. It is indicated that ECV changes in HCM patients without LGE foci may be more subtle [23]. Approximately half of patients with HCM have no LGE on MRI [7], which indicates that this technique cannot be the only one used to diagnose cardiac lesions in HCM patients.

Native T1 mapping is one of the most rapidly developing fields in cardiac magnetic resonance and is of particular benefit in diffuse or early myocardial diseases, where traditional CMR methods such as LGE might fail. The technique is advantageous in patients with renal dysfunction and severe allergic reactions because it precludes the injection of a contrast medium [12,22]. Cardiac myocytes in HCM are hypertrophied, disorganized, and separated by areas of interstitial fibrosis at a cellular level. Native T1 is a composite signal of both the cellular and extracellular components of the myocardium, and because of that, it does not differentiate cellular hypertrophy from diffuse fibrosis. ECV is a more specific assessment of the extracellular matrix alone and therefore a measure of interstitial fibrosis [10]. The ECV values of the whole LV myocardium, ECV of the LV basal layers, ECV of the middle LV layers, ECV of the LV apical layers, and ECV values in LV segments 1–3, 8 and 13–16 were significantly higher in our study in patients with hypertrophic cardiomyopathy than in the control group. It should be emphasized that this indicates diffuse myocardial fibrosis in HCM patients without LGE foci, which is difficult to demonstrate using CMR sequences other than T1-mapping sequences. There were no statistically significant differences in T1 times between the studied groups in segmental analysis covering the entire LV myocardium. Native T1 time increases with aging, interstitial edema secondary to infarction with associated cellular destruction, and enlarged interstitial space from fibrosis [10]. In previous studies, myocardial T1 native time was elevated in patients with hypertrophic cardiomyopathy regardless of the presence or absence of LGE, but T1 values in segments with LGE were significantly higher than those measured in segments without LGE [20]. In one of the studies, patients were 10 years older than the controls, which potentially contributed to this difference [21]. In our study, both groups had a similar mean age.

Native T1 time can be prolonged in many other myocardial pathologies (acute coronary syndromes, myocardial infarction, myocarditis, amyloidosis) and other diseases (increased intracellular water volume in diabetic adults with normal ECV values) [24]. Therefore, specific T1 time thresholds for the detection of pathologies within the myocardium will be able to improve differential diagnosis in the future [22]. These factors, recognized at the molecular level, may have contributed to the differences in T1 native time in previous studies between the HCM and control groups.

In one of the previous studies, T1 time was calculated by one-point sampling mid-cavity segments of the interventricular septum, while whole-heart T1 mapping should be recommended for the accurate assessment of myocardial T1 abnormality in patients with HCM. The single-slice approach might miss a substantial number of segments with elevated native T1 time in patients with HCM. Further multicenter studies are needed to determine the importance of native T1 mapping in HCM patients [21].

Concluding from the T1 native time and other parameters compared in both groups in this study (no changes in left ventricular ejection fraction or left atrial dimensions; no difference in left ventricular end-systolic and end-diastolic diameter; no focal injury in LGE; higher myocardial ECV values), myocardial fibrosis was present and diffuse but not advanced.

Patients with hypertrophic cardiomyopathy in our studied group had an early form of the disease and subtle changes in cardiac tissue (disseminated myocardial fibrosis), which could be detected due to increased ECV values assessed in the T1-mapping sequence.

In view of the eternal aspirations of medicine to detect lesions as early as possible, preferably with the help of non-invasive techniques, the T1-mapping sequence is future-proof, allowing us to provide a group of HCM patients with better risk stratification and care and finally improve their long-term prognosis. In the era of clinical trials of oral antifibrotic agents (e.g., pirfenidone), the prompt diagnosis of hypertrophic cardiomyopathy may contribute to receiving a life-extending therapy. Similarly, quick diagnosis of ATTR amyloidosis and the initiation of treatment with patisiran may be crucial for the prognosis of patients with this type of accumulative disease [25].

The main limitation of this study is the lack of a final confirmation of hypertrophic cardiomyopathy in the examined patients. Myocardial biopsy was not performed in the examined patients. The diagnosis was made based on the entire clinical picture and imaging tests considering other causes of the hypertrophic left ventricular morphotype in the differential diagnosis. All patients in whom amyloidosis, sarcoidosis, athlete’s heart, hypertensive cardiomyopathy, aortic stenosis, third degree obesity, and systemic diseases were considered to be the probable cause of left ventricular hypertrophy were excluded from the study.

## 5. Conclusions

In patients with hypertrophic cardiomyopathy in whom no foci of injury in the left ventricular myocardium were detected in classic CMR sequences, higher ECV values of the left ventricular myocardium were observed.

Advanced left ventricular hypertrophy in patients with hypertrophic cardiomyopathy without myocardium focal injury in the LGE sequence increases the ECV value.

## Figures and Tables

**Table 1:** Clinical characteristics of the study groups.

	HCM Group (n = 28)	CON Group (n = 28)	*p* ^c^
age [years] ^a^	52.17 ± 1.32	51.76 ± 1.19	-
gender ^b^			
men	57.1	57.1	1.00
women	42.9	42.9	1.00
height [cm] ^a^	167.25 ± 1.54	169.00 ± 1.72	-
weight [kg] ^a^	71.14 ± 2.03	73.32 ± 2.17	-
BMI [kg/m^2^] ^a^	25.34 ± 0.71	25.66 ± 0.39	-
overweight/obesity ^b^			
normal body mass	57.1	50.0	0.59
overweight	28.6	39.3	0.40
obesity	14.3	10.7	0.68
coexistence of cardiovascular risk factors ^b^			
arterial hypertension	28.6	32.1	0.77
type 2 diabetes	10.7	17.8	0.45
smoking	39.3	42.8	0.79

a—quantitative variable expressed as mean ± standard error of mean, b—categorical variable expressed as percentage, c—in the case of categorical variables, the *p*-value for the chi-square test; and in the case of quantitative variables, *p*-value for each response variable *p* < 0.05 and the Wilks Lambda value of the one-way MANOVA analysis (Wilks Lambda = 0.938, *p* = 0.506) and for the contrast between CON—control, HCM—hypertrophic cardiomyopathy, BMI—body mass index.

**Table 2:** Standard cardiac magnetic resonance parameters in the study groups.

	HCM Group (n = 28)	CON Group (n = 28)	*p* ^c^
cardiac cavities dimensions			
LAA [cm^2^] ^a^	22.17 ± 1.38	23.42 ± 0.73	-
RAA [cm^2^] ^a^	20.63 ± 0.67	21.74 ± 0.58	-
LVEDD [mm] ^a^	59.76 ± 2.64	57.86 ± 1.66	-
LVESD [mm] ^a^	30.49 ± 2.42	33.13 ± 2.21	-
aIVSDD [mm] ^a^	15.59 ± 0.69	8.47 ± 0.69	0.01
PWDD [mm] ^a^	11.68 ± 0.53	6.96 ± 0.46	0.01
maxLVMDD [mm] ^a^	19.16 ± 0.67	9.14 ± 0.25	0.01
LVM [g] ^a^	215.86 ± 6.39	135.83 ± 7.10	0.01
LVMI [g/m^2^] ^a^	137.49 ± 3.57	71.13 ± 4.72	0.01
thickening of the left ventricular myocardium ^b^			
interventricular septum basal segments	64.3	0.0	0.01
interventricular septum middle segments	60.7	0.0	0.01
interventricular septum apical segments	35.7	0.0	0.01
anterior wall basal segments	46.4	0.0	0.01
anterior wall middle segments	17.8	0.0	0.06
anterior wall apical segments	7.1	0.0	0.15
inferior wall basal segments	3.6	0.0	0.31
inferior wall middle segments	7.1	0.0	0.15
inferior wall apical segments	25.0	0.0	0.01
lateral wall basal segments	10.7	0.0	0.07
lateral wall middle segments	10.7	0.0	0.07
lateral wall apical segments	28.6	0.0	0.01
apex	35.7	0.0	0.01
left ventricular systolic function			
EDV/BSA [ml/m^2^] ^a^	96.18 ± 4.76	95.47 ± 3.60	-
ESV/BSA [ml/m^2^] ^a^	30.57 ± 2.32	30.18 ± 2.17	-
SV/BSA [ml/m^2^] ^a^	62.74 ± 2.13	61.48 ± 2.12	-
EF [%] ^a^	67.15 ± 1.50	66.96 ± 1.12	-
LVSD ^b^	0.0	0.0	1.00
left ventricular morphology			
edema foci ^b^	0.0	0.0	1.00
LGE foci ^b^	0.0	0.0	1.00

a—quantitative variable expressed as mean ± standard error of mean, b—categorical variable expressed as percentage, c—in the case of categorical variables, the *p*-value for the chi-square test; and in the case of quantitative variables, *p*-value for each response variable *p* < 0.05 and the Wilks Lambda value of the one-way MANOVA analysis (Wilks Lambda = 0.075, *p* < 0.01) and for the contrast between CON—control, HCM—hypertrophic cardiomyopathy, aIVSDD—anterior interventricular septal diastolic diameter, BSA—body surface area, EDV—end-diastolic volume, EF—ejection fraction, ESV—end-systolic volume, LAA—left atrial area, LGE—late gadolinium enhancement, LVEDD—left ventricular end-diastolic diameter, LVESD—left ventricular end-systolic diameter, LVM—left ventricular mass, LVMI—left ventricular mass index, LVSD—left ventricular systolic dysfunction, maxLVMDD—maximum left ventricular myocardial diastolic diameter, PWDD—posterior wall diastolic diameter, RAA—right atrial area, SV—stroke volume.

**Table 3:** Myocardial T1 times in the cardiac magnetic resonance T1-mapping sequence in the study groups.

	HCM Group (n = 28)	CON Group (n = 28)	*p* ^b^
whole myocardium [ms] ^a^	1038.15 ± 2.98	1031.56 ± 3.65	-
basal layer [ms] ^a^	1042.56 ± 3.68	1035.84 ± 4.02	-
middle layer [ms] ^a^	1031.11 ± 3.66	1025.17 ± 4.26	-
apical layer [ms] ^a^	1048.64 ± 3.00	1040.97 ± 4.49	-
segment 1 [ms] ^a^	1040.59 ± 4.62	1034.61 ± 4.83	-
segment 2 [ms] ^a^	1048.48 ± 4.38	1039.42 ± 4.18	-
segment 3 [ms] ^a^	1050.03 ± 4.15	1041.58 ± 3.82	-
segment 4 [ms] ^a^	1040.28 ± 4.07	1042.65 ± 3.43	-
segment 5 [ms] ^a^	1037.15 ± 3.96	1037.02 ± 2.99	-
segment 6 [ms] ^a^	1039.12 ± 4.66	1030.62 ± 3.61	-
segment 7 [ms] ^a^	1027.59 ± 3.36	1022.49 ± 3.98	-
segment 8 [ms] ^a^	1039.15 ± 3.79	1032.15 ± 3.84	-
segment 9 [ms] ^a^	1029.13 ± 3.76	1027.58 ± 2.77	-
segment 10 [ms] ^a^	1030.87 ± 3.61	1025.78 ± 3.79	-
segment 11 [ms] ^a^	1034.18 ± 3.79	1026.29 ± 3.77	-
segment 12 [ms] ^a^	1031.84 ± 3.05	1024.42 ± 3.01	-
segment 13 [ms] ^a^	1048.69 ± 3.98	1039.15 ± 3.41	-
segment 14 [ms] ^a^	1049.19 ± 3.74	1040.01 ± 3.59	-
segment 15 [ms] ^a^	1050.24 ± 3.14	1041.03 ± 3.37	-
segment 16 [ms] ^a^	1048.12 ± 3.36	1041.59 ± 4.97	-

a—quantitative variable expressed as mean ± standard error of mean, b—*p*-value for each response variable *p* < 0.05 and the Wilks Lambda value of the one-way MANOVA analysis (Wilks Lambda = 0.947, *p* = 0.589) and for the contrast between CON—control, HCM—hypertrophic cardiomyopathy.

**Table 4:** Extracellular volume (ECV) in the cardiac magnetic resonance T1-mapping sequence in the study groups.

	HCM Group (n = 28)	CON Group (n = 28)	*p* ^b^
whole myocardium [%] ^a^	30.23 ± 0.84	25.08 ± 0.36	0.01
basal layer [%] ^a^	32.14 ± 0.91	26.04 ± 0.73	0.01
middle layer [%] ^a^	28.36 ± 0.80	26.62 ± 0.84	-
apical layer [%] ^a^	31.72 ± 0.51	24.20 ± 0.59	0.01
segment 1 [%] ^a^	35.08 ± 0.69	26.28 ± 0.61	0.01
segment 2 [%] ^a^	34.33 ± 0.78	26.56 ± 0.64	0.01
segment 3 [%] ^a^	33.87 ± 0.53	25.81 ± 0.70	0.01
segment 4 [%] ^a^	29.08 ± 0.75	25.58 ± 0.63	-
segment 5 [%] ^a^	28.86 ± 0.84	25.66 ± 0.72	-
segment 6 [%] ^a^	29.30 ± 0.59	25.90 ± 0.58	-
segment 7 [%] ^a^	27.63 ± 0.56	26.78 ± 0.53	-
segment 8 [%] ^a^	32.17 ± 0.69	26.60 ± 0.63	0.01
segment 9 [%] ^a^	28.48 ± 0.73	26.86 ± 0.63	-
segment 10 [%] ^a^	28.17 ± 0.82	26.02 ± 0.54	-
segment 11 [%] ^a^	27.44 ± 0.85	26.77 ± 0.78	-
segment 12 [%] ^a^	26.39 ± 0.74	26.76 ± 0.42	-
segment 13 [%] ^a^	31.41 ± 0.61	24.03 ± 0.97	0.01
segment 14 [%] ^a^	32.33 ± 0.68	24.97 ± 0.68	0.01
segment 15 [%] ^a^	31.40 ± 0.49	23.94 ± 0.48	0.01
segment 16 [%] ^a^	31.76 ± 0.70	24.05 ± 0.61	0.01

a—quantitative variable expressed as mean ± standard error of mean, b—*p*-value for each response variable *p* < 0.05 and the Wilks Lambda value of the one-way MANOVA analysis (Wilks Lambda = 0.034, *p* < 0.01) and for the contrast between CON—control, HCM—hypertrophic cardiomyopathy.

**Table 5:** The results of the correlation analysis between standard left ventricular evaluation parameters and T1-mapping sequence parameters in cardiac magnetic resonance in the study group.

	Whole Myocardium T1 Time [ms]		Whole Myocardium ECV [%]	
	r	*p*	r	*p*
LVEDD [mm]	0.05	0.84	0.11	0.73
LVESD [mm]	0.07	0.82	0.03	0.94
aIVSDD [mm]	0.17	0.59	0.17	0.59
PWDD [mm]	0.15	0.61	0.18	0.54
maxLVMDD [mm]	0.21	0.22	0.23	0.24
LVM [g]	0.19	0.54	0.19	0.34
LVMI [g/m^2^]	0.21	0.22	0.20	0.22
EDV/BSA [ml/m^2^]	0.14	0.71	0.16	0.49
ESV/BSA [ml/m^2^]	0.02	0.97	0.10	0.56
SV/BSA [ml/m^2^]	0.05	0.84	0.14	0.71
EF [%]	−0.03	0.94	−0.06	0.84

aIVSDD—anterior interventricular septal diastolic diameter, BSA—body surface area, EDV—end-diastolic volume, ECV—extracellular volume, EF—ejection fraction, ESV—end-systolic volume, LVEDD—left ventricular end-diastolic diameter, LVESD—left ventricular end-systolic diameter, LVM—left ventricular mass, LVMI—left ventricular mass index, maxLVMDD—maximum left ventricular myocardial diastolic diameter, PWDD—posterior wall diastolic diameter, SV—stroke volume.

**Table 6:** Results of estimation for the final model obtained using multivariate regression analysis.

Model for Whole Myocardium ECV [%]			
	Intercept	Lvmi [g/m^2^]	Age [Years]
regression coefficient for LVMI < 131.84 g/m^2^	15.528	0.025	0.124
*p*	0.01	0.27	0.04
*p* of model for LVMI < 131.84 g/m^2^	0.19		
regression coefficient for LVMI ≥ 131.84 g/m^2^	24.025	0.038	0.139
*p*	0.01	0.04	0.03
*p* of model for LVMI ≥ 131.84 g/m^2^	0.04		
*p* of whole model	0.03		
determination coefficient (R2)	77.12%		

BMI—body mass index, ECV—extracellular volume, LVMI—left ventricular mass index.

## Data Availability

The data presented in this study are available on request from the corresponding author.
